# Homogeneous Nanostructured VO_2_@SiO_2_ as an Anti-Reflecting Layer in the Visible/Near Infrared Wavelength

**DOI:** 10.3390/ma16176035

**Published:** 2023-09-02

**Authors:** Shuxia Wang, Jiajun He, Panxu Sun

**Affiliations:** 1 School of Materials Science and Engineering, Zhengzhou University of Aeronautics, Zhengzhou 450046, China; shuxiawang1990@yeah.net (S.W.); zhenghang20232023@126.com (J.H.); 2 School of Civil Engineering, Zhengzhou University, Zhengzhou 450001, China

**Keywords:** homogeneous, vanadium oxide, pattern SiO_2_, low-reflecting, visible/near infrared

## Abstract

Low reflectivity is of great significance to photoelectric devices, optical displays, solar cells, photocatalysis and other fields. In this paper, vanadium oxide is deposited on pattern SiO_2_ via atomic layer deposition and then annealed to characterize and analyze the anti-reflection effect. Scanning electron microscope (SEM) images indicate that the as-deposited VO_x_ film has the advantages of uniformity and controllability. After annealing treatment, the VO_2_@pattern SiO_2_ has fewer crevices compared with VO_2_ on the accompanied planar SiO_2_ substrate. Raman results show that there is tiny homogeneous stress in the VO_2_ deposited on pattern SiO_2_, which dilutes the shrinkage behavior of the crystallization process. The optical reflection spectra indicate that the as-deposited VO_x_@pattern SiO_2_ has an anti-reflection effect due to the combined mechanism of the trapping effect and the effective medium theory. After annealing treatment, the weighted average reflectance diminished to 1.46% in the visible near-infrared wavelength range of 650–1355 nm, in which the absolute reflectance is less than 2%. Due to the multiple scattering effect caused by the tiny cracks generated through annealing, the anti-reflection effect of VO_2_@pattern SiO_2_ is superior to that of VO_x_@pattern SiO_2_. The ultra-low reflection frequency domain amounts to 705 nm, and the lowest absolute reflectance emerges at 1000 nm with an astonishing value of 0.86%. The prepared anti-reflective materials have significant application prospects in the field of intelligent optoelectronic devices due to the controllability of atomic layer deposition (ALD) and phase transition characteristics of VO_2_.

## 1. Introduction

Research indicates that 30% of incident light has been wasted due to surface reflection. Since solar energy is a renewable, clean, and safe energy source, [1] and reducing energy loss caused by light reflection is essential to improve conversion efficiency in photovoltaic applications [2]. Accordingly, the preparation and exploration of low-reflection structures have important application prospects in fields such as optoelectronic components, monitors, solar cells [3], infrared imaging, photocatalysis, and electromagnetic stealth [4,5].

According to the λ/4 principle of optical interference, a traditional method is about coating low-refractive-index anti-reflection layer on the glass, which can only reduce the reflectivity within narrow and limited bands [6]. In recent years, extensive research has been conducted on reducing reflectivity, including patterned substrates [7], roughening the surface of the device, photonic crystal regulation technology [8], single/multi-layer anti-reflection coatings [9], biomimetic moth eye structure [10,11], and surface microtexturing technology [12], all of which have achieved lower reflectance. Kim et al. [13] used SiO and SiNx as single-layer anti-reflective coatings to improve the efficiency of solar cells by 10% and 25%, respectively. Gao et al. [14] prepared a uniform periodic anti-reflection structure on flexible solar cell thin films, reducing its reflectivity to 2.6%. Polman et al. [15] designed a two-dimensional periodic array of subwavelength silicon nanocylinders as Mie resonances with strong substrate coupling, producing almost zero total reflectance throughout the entire spectral range from ultraviolet to near-infrared. Yan et al. [16] induced nanoscale composite material structures with nano textures on the surface of glass via an infrared femtosecond laser method. The average reflectivity decreased by 45.5% in the wavelength range of 300–800 nm compared to untreated glass. Barreda et al. [17] increased the radiation scattered into a silicon photovoltaic wafer by combining nanoparticles and multilayer system, which can improve the utilization of scattered light absorption while reducing reflection simultaneously. Li et al. [9] obtained an average reflectivity of only 1.59% in the wavelength range of 300–900 nm using a four-layer anti-reflection coating of MgF_2_/ZnS/MgF_2_/ZnS. Yang et al. [18] constructed high-aspect-ratio nanowire microstructures modified with metal nanoparticles on a silicon surface by means of laser direct writing technology. The reflection was reduced to less than 1% in the range of 300–1200 nm, profiting from the combined mechanism of light capture and plasma resonance effect. Ren et al. [19] demonstrated that the average reflection loss of 1% in the mid infrared region can be obtained based on the graphene plasma circuit with a biomimetic moth-eye structure.

The preparation methods of the as-mentioned anti-reflection structures mainly include sol–gel, vapor deposition, impregnation, sputtering, nanoimprinting [11], direct hot pressing [20], electrospinning, laser processing [18], photolithography, and the template method [21]. These technical methods are relatively mature and widely used, while having certain limitations in with respect to the precise control of material thickness on profiled structures. As an innovative deposition technology with a self-limiting growth mechanism, atomic layer deposition can achieve thickness controllability on the atomic level and a uniform coating effect on various profiled structures with good compatibility [22]. It avoids the issue of poor combination of materials with substrates prepared via the coating or impregnation method, as well as the selective defects of nanoimprinting, photolithography, and other technologies, which is expected to have significant influence in future microelectronic devices and other fields [23]. 

Vanadium dioxide is an intelligent phase transition material with a reversible metal-insulator phase transition at the intrinsic temperature (about 68 °C) accompanied by an abrupt change in electrical and optical properties [24]. When the ambient temperature is higher than the phase transition temperature, it exhibits optical reflection characteristics; otherwise, it represents optical transmission characteristics, which have significant applications in fields such as smart windows in reducing energy consumption [25], laser protection, and optical switches [26]. The preparation of VO_2_ from metal complexes is a relatively mature technique, which is easy to implement [27]. The most commonly used antireflective materials include SiO_2_ [11], MgF_2_, TiO_2_, Al_2_O_3_, Si_3_N_4_, ZrO_2_, and other oxides or nitrides [28], which possess well compatibility with silicon [29] and exhibit stable performance [30] in semiconductor photovoltaic devices. The phase transition characteristics of vanadium dioxide can be effectively controlled by introducing an anti-reflection structure. Lee et al. [31,32] indicated that the thermochromism characteristics of VO_2_ can be promoted by depositing anti-reflection coating SiO_2_ using magnetron sputtering. Okimura et al. [33] improved the luminous transmittance and solar modulation of VO_2_-based smart windows by SiO_2_ antireflection coatings. On the contrary, if vanadium dioxide is introduced into the anti-reflection system, an intelligent anti-reflection structure that changes with temperature can be obtained because of its reversible effect on light before and after phase transition [34]. However, research in this field is relatively scarce.

In this paper, a vanadium oxide layer is deposited on the surface of pattern silicon dioxide substrate via atomic layer deposition. The experimental results show that the anti-reflection characteristics of the composite structure have been improved with low-weighted-average reflectivity and low absolute reflectivity at specific wavelengths.

Furthermore, the result is expected to provide a significant opportunity for its application and development in the field of intelligent anti-reflection due to the intelligent phase transition characteristics of vanadium dioxide. Predictably, when the temperature is lower than the phase transition temperature of VO_2_, the prepared VO_2_@pattern SiO_2_ exhibits a low reflection effect. When the temperature is higher than that of VO_2_ to make it transform from a semiconductor phase to a metal phase, the prepared VO_2_@pattern SiO_2_ material may exhibit a total reflection effect, which can be applied to intelligent optical devices.

## 2. Materials and Methods

### 2.1. Subsection Synthesis of Vanadium Oxide@Pattern SiO_2_ Nanostructure

**Figure 1:**
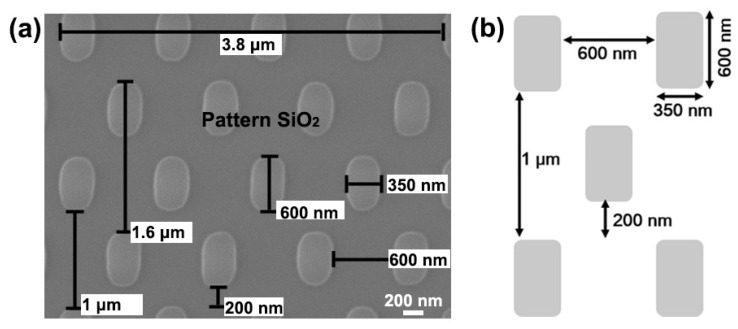
Structural parameter diagram of pattern SiO_2_: (**a**) the SEM image; (**b**) diagrammatic sketch.

The substrates of planar SiO_2_ and pattern SiO_2_ are hydrophilically treated. The structural parameter image and diagram of pattern SiO_2_ are shown in Figure 1. Specifically, the substrates are immersed in acetone solution, anhydrous ethanol solution, and a solution with a ratio of H_2_O_2_ and H_2_SO_4_ of 3:1 for 20 min, sequentially. Then, the substrates are flushed with deionized water and anhydrous ethanol, respectively. 

Subsequently, the substrates are purged with a nitrogen gas flow before being put into the atomic layer deposition (ALD) chamber (Picosun R200, Finland) at 200 °C. The precursors of vanadium oxytriisopropoxide (VO(OC_3_H_7_)_3_, VTIP) and deionized water are pulsed into the reactor to deposit vanadium oxide. The source cylinders containing VTIP and H_2_O are set as 60 °C and room temperature, respectively. The pulse times are 1.6 s and 0.1 s, and the purge time are 12 s and 10 s. The carrier gas flow rates of N_2_ are 150 sccm, and the total cycles are set to 3500, which is approximately 55 nm.

**Figure 2:**
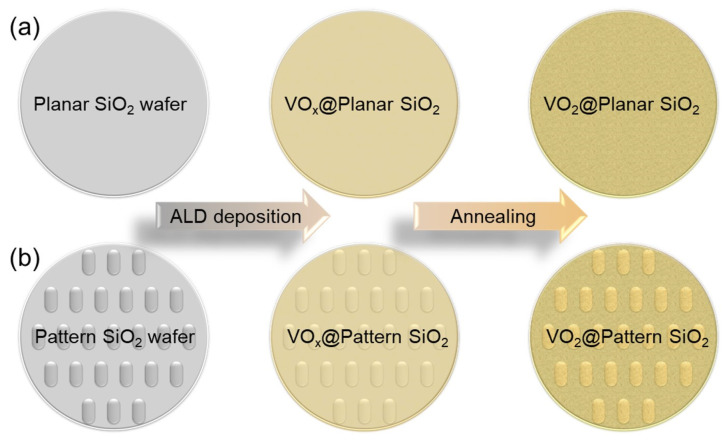
Schematic diagram of the preparation processes of vanadium oxide@planar/pattern SiO_2_ nanostructure: (**a**) the accompanying planar SiO_2_ substrate; (**b**) the pattern SiO_2_ substrate.

Ultimately, the as-prepared vanadium oxide@planar/pattern SiO_2_ thin films are annealed at 550 °C for 4 h. The ramp rate of the annealing furnace is 1 °C/min, and the gas flow rate of Ar is 50 sccm. During the annealing process, the pressure of the furnace is stabilized at 4.0 mbar by manipulating the vacuum pump. The schematic diagram of the preparation processes of vanadium oxide@planar/pattern SiO_2_ nanostructure is shown in Figure 2.

### 2.2. Characterization

The surface morphology and cross-sectional structure of the samples are characterized via scanning electron microscopy (SEM Sirion 200, FEI company, Hillsboro, OR, USA). The feature peak position information of VO_2_ deposited on planar/pattern SiO_2_ is characterized via Raman scanning using a Nanofinder 30 (TII Tokyo Instruments, Tokyo, Japan) equipped with an excitation laser of 532 nm. The stress distribution information of the VO_2_@pattern SiO_2_ nanostructure is conducted via Raman mapping scanning with a step of 100 nm and a spectral resolution of less than 1 cm^−1^. The scanning area is 4 × 5 μm. The reflectivity spectra are performed in a 5 nm step by UV/VIS/NIR spectrometer Lambda 950 (Perkin Elmer, Waltham, MA, USA) with wavelengths ranging from 250 to 2500 nm.

## 3. Results and Discussion

VO_x_@planar/pattern SiO_2_ thin films are obtained via hydrophilic treatment and ALD procedure while VO_2_@planar/pattern SiO_2_ nanostructures are acquired via a post-annealing process. Figure 2 shows the schematic diagram of the preparation processes, in which Figure 2a displays the accompanying planar SiO_2_ substrate and Figure 2b represent the pattern SiO_2_ substrate. 

**Figure 3:**
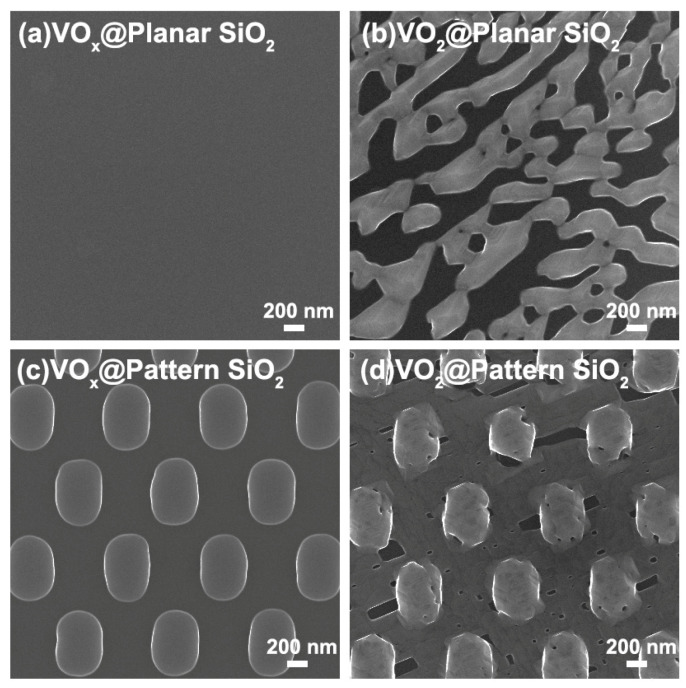
Top-view SEM images of vanadium oxide@planar/pattern SiO_2_ nanostructure: (**a**) the surface image of VO_x_@planar SiO_2_ substrate before the annealing process; (**b**) the surface image of VO_2_@planar SiO_2_ substrate after the annealing process; (**c**) the surface image of VO_x_@pattern SiO_2_ substrate before the annealing process; (**d**) the surface image of VO_2_@pattern SiO_2_ substrate after the annealing process.

Figure 3 demonstrates the top-view SEM images of vanadium oxide@planar/pattern SiO_2_ nanostructures. Figure 3a shows a smooth surface of amorphous VO_x_ film directly deposited via ALD procedure on the accompanying planar SiO_2_ substrate. After post annealing treatment, the sample of amorphous VO_x_@SiO_2_ turns into crystallized VO_2_@SiO_2_. Moreover, irregular stripes and large cracks caused by the shrinkage behavior during the crystallization process appear, just as displayed in Figure 3b. For amorphous VO_x_ deposited on pattern SiO_2_ substrate, the original architecture is well maintained with an obvious and unified increase in thickness, as shown in Figure 3c. The uniform morphology of primordial VO_x_@pattern SiO_2_ film certifies that the film deposited by ALD can achieve a full coverage effect in various heterotypic structures. After the annealing process, the amorphous VO_x_@pattern SiO_2_ smooth film converts into a crystallized VO_2_@pattern SiO_2_ nanostructure. Cracks also emerge due to the contractions of the crystallization process, but the crevices are far smaller than those of VO_2_@planar SiO_2_. It can be inferred that there is a tenuous stress between the interface of VO_2_ and pattern SiO_2_ which prevents the severe shrinkage behavior during the crystallization process. 

**Figure 4:**
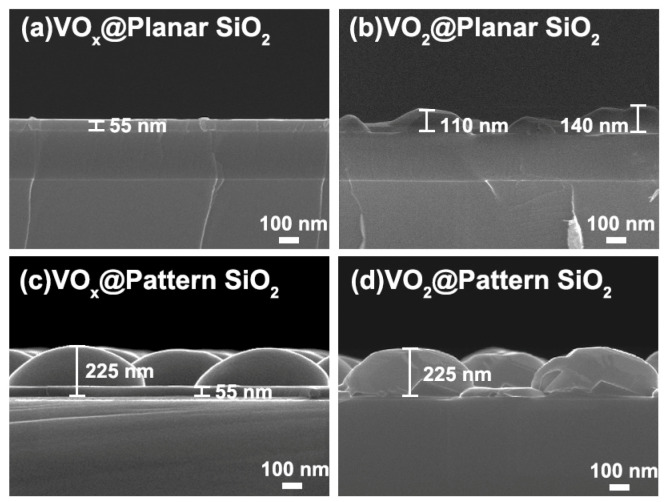
Cross-section SEM images of vanadium oxide@planar/pattern SiO_2_ nanostructure: (**a**) the fracture surface of VO_x_@planar SiO_2_ substrate before the annealing process; (**b**) the fracture surface of VO_2_@planar SiO_2_ substrate after the annealing process; (**c**) the fracture surface of VO_x_@pattern SiO_2_ substrate before the annealing process; (**d**) the fracture surface of VO_2_@pattern SiO_2_ substrate after the annealing process.

Figure 4 shows the cross-sectional morphologies of the samples, which further certify the film’s uniformity prepared by atomic layer deposition, as well as full cladding of the dysmorphism architecture. Figure 4a displays the fracture surface of VO_x_@SiO_2_ with a uniform thickness of approximately 55 nm, indicating a deposition rate of 0.016 nm/cycle. After the annealing process, the VO_2_ crystal stripe shows heterogeneous morphology with a thickness of approximately 100 nm, just as shown in Figure 4b. For the sample of VO_x_@pattern SiO_2_, the periodic structure of pattern SiO_2_ is well preserved and has equal altitude. The thickness of VO_x_ on pattern SiO_2_ is 55 nm, which is consistent with that on planar SiO_2_, indicating that the deposition rate is only related to the source material and has no selectivity towards the basement structure. The gauge of pattern SiO_2_ does not change significantly before and after annealing; both are approximately equal to 225 nm, as revealed in Figure 4d. It is notable that the height of pattern SiO_2_ is basically homogeneous, which means that the VO_2_ still maintains a uniform thickness after the crystallization procedure due to the stress between the interface of crystal VO_2_ and pattern SiO_2_. 

**Figure 5:**
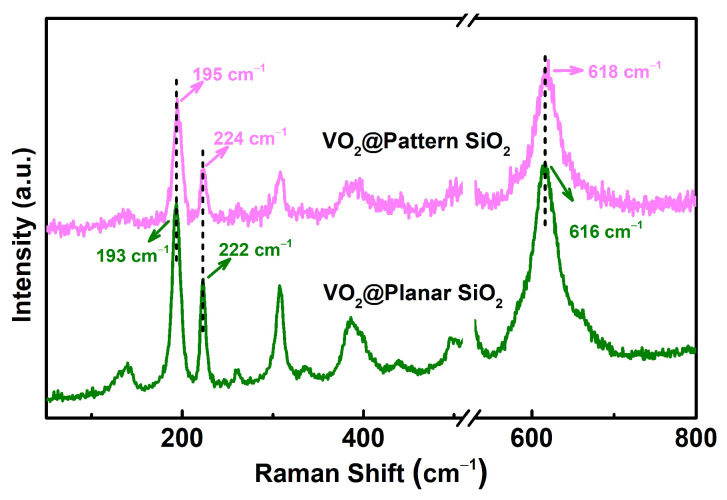
Raman spectra of VO_2_@planar SiO_2_ (olive line) and VO_2_@pattern SiO_2_ (light magenta line).

The Raman spectra confirm the existence of the stress, as shown in Figure 5. After the annealing process, the specimen reveals typical characteristic peaks of VO_2_ (olive line); the peaks located at 193 and 222 cm^−1^ represent V-V dimer vibration, with the peak at 616 cm^−1^ being the vibration peak of the V–O bond. Compared to the sample of VO_2_@planar SiO_2_, the Raman peaks of VO_2_@pattern SiO_2_ show a tiny blue shift (light magenta line), with characteristic peaks of 195, 224, and 618 cm^−1^, respectively, indicating the existence of internal stress on the interface about VO_2_ and the pattern SiO_2_ substrate. The Raman shift reasonably explains the appearance of trace surface shrinkage of VO_2_@pattern SiO_2_ during the annealing crystallization process.

**Figure 6:**
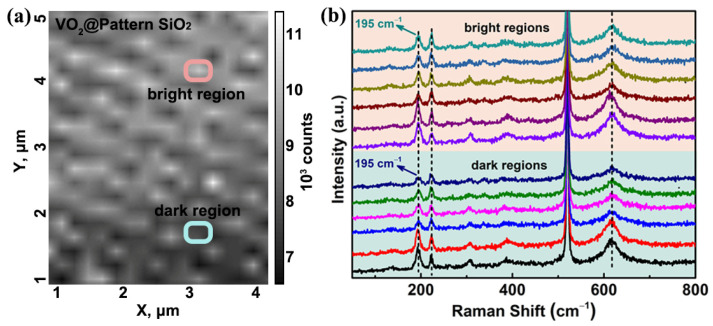
Raman surface scanning of VO_2_@pattern SiO_2_ with an area of 4 × 5 μm: (**a**) intensity map at a Raman shift of 500 cm^−1^; the bright region enclosed within the gouache frame represents the protruding parts of the VO_2_@pattern SiO_2_, the dark region in aqua frame symbolizes the plane zone on the contrary; (**b**) the in situ Raman spectra of the bright and dark area marked in the Raman intensity mapping.

Since it is well known that the phenomenon of stress concentration is unfavorable for the application of materials in fields such as optical displays and electronic components. Whether the stress is homogeneous on the sample of VO_2_@pattern SiO_2_ is rather essential and should be eliminated as possible. Further Raman surface scanning characterization with an area of 4 × 5 μm is performed in order to characterize whether stress concentration is present, just as shown in Figure 6. Figure 6a displays the intensity map at a Raman shift of 500 cm^−1^, in which the periodic structure of the VO_2_@pattern SiO_2_ can be identified due to the various focusing heights on the surface. The bright regions represent the protruding parts of the VO_2_@pattern SiO_2_, such as the section enclosed within the gouache frame, while the dark regions, as shown in the aqua frame, symbolize the plane zone on the contrary. By comparing the Raman spectra extracted randomly from selected points in the bright and dark regions as displayed in Figure 6b, it is found that the Raman offset occurred simultaneously on the entire sample surface without any difference. Therefore, there is no stress concentration phenomenon, and the stress distribution is uniform at the entire surface of VO_2_@pattern SiO_2_ sample. The homogenous stress distribution is of great significance for the limpid imaging of optical displays and the controllable preparation of optoelectronic components.

**Figure 7:**
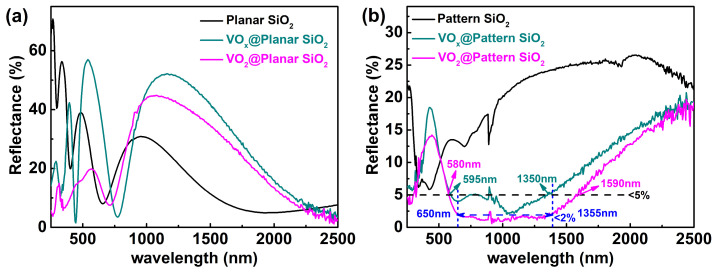
Reflectance spectra of vanadium oxide@planar/pattern SiO_2_ nanostructure: (**a**) the spectra of the planar SiO_2_ substrate (black line), the VO_x_@planar SiO_2_ substrate before the annealing process (dark cyan line), and the VO_2_@planar SiO_2_ substrate after the annealing process (magenta line); (**b**) the spectra of the pattern SiO_2_ substrate (black line), the VO_x_@pattern SiO_2_ substrate before the annealing process (dark cyan line), and the VO_2_@pattern SiO_2_ substrate after the annealing process (magenta line).

Figure 7 displays the reflectance spectra of the mentioned samples. According to *nd* = λ/4 and *nd* = λ/2 regulation, the wavelength positions of the minimum and maximum reflectivity will shift red as the film increases. It is reasonable that the reflection curve of planar SiO_2_ has an apparent λ/4 interference cancellation wave spectrum, as shown in Figure 7a with black line. With the deposition of 55 nm VO_x_ layer, the thickness of VO_x_@planar SiO_2_ increases, which will directly affect the reflection pattern. The reflectance of VO_x_@planar SiO_2_ with the minimum value shows a red shift compared to the sample of planar SiO_2_, as displayed in Figure 7a with dark cyan line. The phenomenon matched well with λ/4 principle, which further confirm that the as-deposited VO_x_ film on planar SiO_2_ is rather uniform and compact. After the annealing process, the reflectance of sample VO_2_@planar SiO_2_ is different from that of VO_x_@planar SiO_2_, as shown in Figure 7a with a magenta line. The reason is that a large number of irregular striped cracks appeared on the surface of the film after the annealing treatment, which can increase the optical path to a certain extent. For the reflection spectrum in the high-frequency band, the samples with grooved shapes reveal lower reflectivity. It is reasonable that multiple scattering mechanism plays a dominant factor in the low-wavelength band due to the fact that the incident wavelength is equivalent to the dimensions of stripe crevice; while in the low-frequency band, the samples of VO_2_@planar SiO_2_ and VO_x_@planar SiO_2_ exhibit similar reflectivity, as shown in Figure 7a between 700 nm and 2500 nm. The multiple scattering mechanism no longer affects reflectivity since the incident wavelength is much longer than the dimensions of the groove. The vanadium oxide on the surface of planar SiO_2_ can be seen as an effective dielectric layer at this point, and the surface reflection is related to its effective refractive index, which shows no significant difference between VO_x_ and VO_2_ materials.

For samples deposited on pattern SiO_2_ substrate, the reflectance shows great difference with the specimens that deposited on planar SiO_2_ as shown in Figure 7b. The anti-reflection outcome is mainly because of the dominating trapping effect caused by the pattern structure. The mechanism of the light trapping effect is well known to make some protrusions or pits on the surface to increase the optical path of light and increase the absorption of photons to reduce the reflectivity of the sample. When the sample is set to a periodic array structure with concave and convex surfaces, incident light will be dispersed at various angles through reflection, refraction, and scattering, and the confined light wavelength can be manipulated by regulating the morphology of the light trapping structure. The reflectance spectrum of pure pattern SiO_2_ substrates indicates that the λ/4 regulation disappears due to the fluctuation of height, as shown in Figure 7b with a black line, and its reflectivity is below 26% throughout the entire spectral range. The sample of VO_x_@pattern SiO_2_ shows an excellent anti reflection effect, with an absolute reflectance of less than 5% in the visible and near-infrared bands of 595–1350 nm, as displayed in Figure 7b with a dark cycle line. The low-reflection wavelength band is up to 755 nm. The low reflectivity at this point can be explained by the effective medium theory, which means that the as-deposited VO_x_ on pattern SiO_2_ causes a refractive index gradient in the direction of the incident light. As a transition metal oxide, vanadium has various oxide forms with different stoichiometric ratios, and its valence states can vary from +1 to +5. In the case of metal oxides, departure from stoichiometry may lead to metal-phase defects and increased optical absorption [35]. Further, our previous research literature indicates that pure vanadium oxides with single valence states are difficult to obtain [36]. It means that the as-deposited VO_x_ are essentially composed of various valence states of vanadium oxides, which processing gradients of refractive index. This provides a theoretical basis for the small continuous changes in the refractive index of the vanadium oxide dielectric layer, weakening the reflection loss caused by the sudden change in the refractive index of light from the air to the sample. Thus, the sample of VO_x_@pattern SiO_2_ has an excellent anti-reflection effect, as shown in Figure 7b with a dark cycle line. In other words, the sample of VO_x_@pattern SiO_2_ exhibits a significant anti-reflection effect, profiting from the combined mechanism of the trapping effect and the effective medium theory. 

After annealing, the reflectivity of sample VO_2_@pattern SiO_2_ was optimized further, as displayed in Figure 7b with a magenta line. The absolute reflectivity is less than 5% in the visible near-infrared band of 580–1590 nm, and the low reflection frequency domain is as high as 1010 nm. While the absolute reflectivity in the 650–1355 nm band is even less than 2%, as shown in the blue dashed line of Figure 7b, the ultra-low reflection frequency domain amounts to 705 nm. Furthermore, the absolute reflectance achieved an astonishing 0.86% at a wavelength of 1000 nm. Except for the combined original trapping effect and the effective medium theory mechanism, the further reduction of reflectivity benefited from the multiple scattering mechanism caused by the tiny holes appearing on the surface of the annealed VO_2_ film. More clearly, although the refractive index and other physical properties of VO_x_ and VO_2_ are rather similar, the small holes that appear on the surface of VO_2_ after the annealing treatment increase the optical path, providing an advantageous condition for reducing the reflectivity of incident light on the surface due to the multiple scattering mechanism in the crevices.

In order to quantitatively evaluate the anti-reflection effect caused by vanadium oxide deposition, the weighted average reflectance and anti-reflection efficiency of the reflection spectra in Figure 7b, considering the wavelength from 580 to 1590 nm, which has an absolute reflection of less than 5%, has been calculated using the following Equations (1) and (2) [37]:

(1) $$R_{W}=\frac{\int_{580}^{1590}R(\lambda )\Phi (\lambda )d\lambda }{\int_{580}^{1590}\Phi (\lambda )d\lambda }$$

(2) $$R_{anti}=\frac{100-R_{W}}{100}\times 100\%,$$

where R(λ) is the reflectance derived from Figure 7b, and Φ(λ) is the spectral irradiance of AM 1.5G. 

The specific values of vanadium oxide@pattern SiO_2_ are revealed in Table 1, including the weighted average reflectance (*R_w_*), the reduced *R_w_* over pattern SiO_2_, the anti-reflection efficiency (*R_anti_*), and the improved *R_anti_* over pattern SiO_2_.

The weighted average reflectance of VO_x_@pattern SiO_2_ achieved 4.41%, which is 74.1% lower than that of pattern SiO_2_. The anti-reflection efficiency is as high as 95.6%, which is 15.2% higher than that of pattern SiO_2_. This phenomenon effectively proves that the as-deposited VO_x_ has a certain anti-reflection effect as an effective medium on pattern SiO_2_, while having no effect on planar SiO_2_, where the λ/4 interference dominates the mechanism. The anti-reflection effect of VO_2_@pattern SiO_2_ has been further improved with a weighted average reflectance of 1.80%, which is 89.4% lower than that of pattern SiO_2_ and much lower than that of VO_2_@pattern SiO_2_. This result is consistent with the previous analysis that the small cracks on the surface of VO_2_ during the annealing process are beneficial for further reduction of reflectivity due to the multiple scattering mechanism. The anti-reflection efficiency amounts to 98.2%, which is 18.3% above that of patterned SiO_2_.

Moreover, the absolute reflection of VO_x_@pattern SiO_2_ in wavelength from 650 to 1355 nm is less than 2%, as displayed in Table 2. The weighted average reflectance of VO_x_@pattern SiO_2_ achieved 4.13%, which is 76.1% lower than that of pattern SiO_2_. The anti-reflection efficiency is as high as 95.9%, which is 16.0% higher than that of pattern SiO_2_. Furthermore, the anti-reflection effect of VO_2_@pattern SiO_2_ has been enhanced with a weighted average reflectance of 1.46%, which is 91.6% lower than that of pattern SiO_2_. The anti-reflection efficiency is approximately to 98.5%, which is 19.1% higher than that of patterned SiO_2_.

## 4. Conclusions

The samples of vanadium oxide@pattern SiO_2_ prepared via atomic layer deposition have a uniform film surface, homogeneous stress distribution, controllable preparation conditions and methods, low weighted average reflectivity, and excellent anti-reflection efficiency in the visible and near-infrared wavelengths. The sample of VO_x_@pattern SiO_2_ has an absolute reflectance of less than 5% in the range of 595–1350 nm, with a reduced reflection frequency domain of 755 nm, and the minimum absolute reflectance of 2.06% appears at 1035 nm. The absolute reflectance of VO_2_@pattern SiO_2_ is less than 5% in the range of 580–1590 nm with low reflection domain amounting to 1010 nm. And the absolute reflectance in the range of 650–1355 nm is less than 2% with an ultra-low reflection domain of 705 nm. The minimum absolute reflectance of 0.86% emerged at 1000 nm. Further calculations indicate that the weighted average reflectance of the as-deposited VO_x_@patterned SiO_2_ decreased by 74.1% compared to patterned SiO_2_ in the range of 580–1590 nm (from 17.0% to 4.41%), and the anti-reflection efficiency increased by 15.2% (from 83.0% to 95.6%). The weighted average reflectance of VO_2_@pattern SiO_2_ after annealing decreased by 89.4% (from 17.0% to 1.80%), and the anti-reflection efficiency increased by 18.3% (from 83.0% to 98.2%). When the anti-reflection band of 650–1355 nm is considered, the VO_2_@pattern SiO_2_ has an absolute reflectance of less than 2%. The weighted average reflectance of the as-mentioned samples further decreased from 17.3% to 4.13% and 1.46%, with decreased rate of 76.1% and 91.6%, respectively. In addition, the anti-reflection efficiency increased from 82.7% to 95.9% and 98.5% with an increase of 16.0% and 19.1%, respectively.

As to future prospects, the vanadium oxide@pattern SiO_2_ structure is expected to provide referential value for the application of intelligent anti-reflection devices that change with the circumstance. The theoretical foundation is that the optical performance of vanadium oxide changes abruptly from optical transmission to optical reflection due to the phase transition characteristics at approximately room temperature.

## Figures and Tables

**Table 1:** Weighted average reflectance and anti-reflection efficiency of the reflection spectra in Figure 7b considering the wavelength from 580 to 1590 nm, and an absolute reflection is less than 5%.

Samples	*R_w_* (%)	Reduced *R_w_* over Pattern SiO_2_ (%)	*R_anti_* (%)	Improved *R_anti_* over Pattern SiO_2_ (%)
Pattern SiO_2_	17.0		83.0	
VO_x_@pattern SiO_2_	4.41	74.1	95.6	15.2
VO_2_@pattern SiO_2_	1.80	89.4	98.2	18.3

**Table 2:** Weighted average reflectance and anti-reflection efficiency of the reflection spectra in Figure 7b considering the wavelength from 650 to 1355 nm, and an absolute reflection is less than 2%.

Samples	*R_w_* (%)	Reduced *R_w_* over Pattern SiO_2_ (%)	*R_anti_* (%)	Improved *R_anti_* over Pattern SiO_2_ (%)
Pattern SiO_2_	17.3		82.7	
VO_x_@pattern SiO_2_	4.13	76.1	95.9	16.0
VO_2_@pattern SiO_2_	1.46	91.6	98.5	19.1

## Data Availability

Some or all data that support the findings of this study are available from the corresponding author upon reasonable request.

## References

[1] Helveston J.P., He G., Davidson M.R. (2023). Quantifying the cost savings of global solar photovoltaic supply chains. Nature.

[2] Hassan S., Dhimish M. (2022). Review of current state-of-the-art research on photovoltaic Soiling, anti-reflective coating, and solar roads deployment supported by a pilot experiment on a PV Road. Energies.

[3] Ito S., Murakami T.N., Comte P., Liska P., Grätzel C., Nazeeruddin M.K., Grätzel M. (2008). Fabrication of thin film dye sensitized solar cells with solar to electric power conversion efficiency over 10%. Thin Solid Film..

[4] Pendry J.B., Schurig D., Smith D.R. (2006). Controlling electromagnetic fields. Science.

[5] Leonhardt U., Tyc T. (2009). Broadband invisibility by non-Euclidean cloaking. Science.

[6] Priyadarshini B.G., Sharma A.K. (2016). Design of multi-layer anti-reflection coating for terrestrial solar panel glass. Bull. Mat. Sci..

[7] Zäll E., Järn M., Karlsson S., Tryggeson H., Tuominen M., Sundin M., Wågberg T. (2023). Aerosol-based deposition of broadband antireflective silica coating with closed mesoporous structure. Sol. Energy Mater. Sol. Cells.

[8] Kim K.H., Park Q.H. (2013). Perfect anti-reflection from first principles. Sci. Rep..

[9] Valiei M., Shaibani P.M., Abdizadeh H., Kolahdouz M., Asl Soleimani E., Poursafar J. (2022). Design and optimization of single, double and multilayer anti-reflection coatings on planar and textured surface of silicon solar cells. Mater. Today Commun..

[10] Huang Y., Chattopadhyay S., Jen Y., Peng C., Liu T., Hsu Y., Pan C., Lo H.C., Hsu C., Chang Y. (2007). Improved broadband and quasi-omnidirectional anti-reflection properties with biomimetic silicon nanostructures. Nat. Nanotechnol..

[11] Huh D., Shin J.H., Byun M., Son S., Jung P.H., Choi H.J., Kim Y.D., Lee H. (2017). Analysis of long-term monitoring data of PV module with SiO_x_-based anti-reflective patterned protective glass. Sol. Energy Mater. Sol. Cells.

[12] Chattopadhyay S., Huang Y., Jen Y., Ganguly A., Chen K., Chen L. (2010). Anti-reflecting and photonic nanostructures. Mat. Sci. Eng. R.

[13] Lee E.G., Kim J.H., Ko H., Kim C.Y. (2015). The anti-reflection coating using the silicon nitride and silicon monoxide for InP based solar cells. J. Comput. Theor. Nanosci..

[14] Chen F., Zhan X., Gao M., Tie S., Gao W. (2017). Anti-reflective microstructure array and its performance evaluation in thin film flexible solar cells. Mod. Phys. Lett. B.

[15] Spinelli P., Verschuuren M.A., Polman A. (2012). Broadband omnidirectional antireflection coating based on subwavelength surface Mie resonators. Nat. Commun..

[16] Yin J., Yan H., Gesang D., Wang R., Cao S., Zhou R., Li Y. (2022). General strategy toward laser single-step generation of multiscale anti-reflection structures by marangoni effect. Micromachines.

[17] Barreda Á., Albella P., Moreno F., González F. (2021). Broadband unidirectional forward scattering with high refractive index nanostructures: Application in solar cells. Molecules.

[18] Yang J., Luo F., Kao T., Li X., Ho G., Teng J., Luo X., Hong M. (2014). Design and fabrication of broadband ultralow reflectivity black Si surfaces by laser micro/nanoprocessing. Light-Sci. Appl..

[19] Zhu B., Ren G., Cryan M.J., Gao Y., Li H., Wang Q., Wan C., Jian S. (2015). Biomimetic ‘moth-eye’ anti-reflection boundary for graphene plasmons circuits. J. Opt..

[20] Li X., Wu M., Chen J., Zhou X., Ren Q., Wang L., Shen B., Zheng W. (2023). A facile and large-scale approach to prepare macroscopic segregated polyether block amides/carbon nanostructures composites with a gradient structure for absorption-dominated electromagnetic shielding with ultra-low reflection. Compos. Commun..

[21] Vu T.D., Cao X., Hu H., Bao J., Cao T., Hu J., Zeng X., Long Y. (2021). A universal robust bottom-up approach to engineer Greta-oto-inspired anti-reflective structure. Cell Rep. Phys. Sci..

[22] D’Acunto G., Shayesteh P., Kokkonen E., Boix de la Cruz V., Rehman F., Mosahebfard Z., Lind E., Schnadt J., Timm R. (2023). Time evolution of surface species during the ALD of high-k oxide on InAs. Surf. Interfaces.

[23] Jones J.C., Delegan N., Heremans F.J., Martinson A.B.F. (2023). Nucleation dependence of atomic layer deposition on diamond surface termination. Carbon.

[24] He Z., Qi Z., Yang B., Lu P., Shen J., Dilley N.R., Zhang X., Wang H. (2023). Controllable phase transition properties in VO_2_ films via metal-ion intercalation. Nano. Lett..

[25] Liao S., Wang X., Huang H., Shi Y., Wang Q., Hu Y., Zhu P., Sun R., Wong C., Wan Y. (2023). Intelligent shielding material based on VO_2_ with tunable near-field and far-field electromagnetic response. Chem. Eng. J..

[26] Calvi L., van Zandvoort R., Leufkens L., Hupperetz J.F.B., Habets R., Mann D., Meulendijks N., Verheijen M.A., Elen K., Hardy A. (2023). Thermochromic glass laminates comprising W/VO_2_ nanoparticles obtained by wet bead milling: An in-depth study of the switching performance. Sol. Energ. Mat. Sol. C..

[27] Lv X., Cao Y., Yan L., Li Y., Song L. (2017). Atomic layer deposition of VO_2_ films with Tetrakis-dimethyl-amino vanadium (IV) as vanadium precursor. Appl. Surf. Sci..

[28] Sarkın A.S., Ekren N., Sağlam Ş. (2020). A review of anti-reflection and self-cleaning coatings on photovoltaic panels. Sol. Energy.

[29] Shafique R., Latif H., Sattar A., Shabbir S.A. (2023). Effect of anti-reflecting layers on device performance of SWCNTs/Si hetero-junction hybrid solar cells. Opt. Mater..

[30] Manna S., Adak D., Manna S., Maity S., Jana S., Bhattacharya R., Medda S.K. (2023). Antireflection cum photocatalytic with superhydrophilic based durable single layer mesoporous TiO_2_-ZrO_2_ coating surface for efficient solar photovoltaic application. Sustain. Energy Techn..

[31] Lee M.H., Cho J.S. (2000). Better thermochromic glazing of windows with anti-reflection coating. Thin Solid Film..

[32] Lee M.H. (2002). Thermochromic glazing of windows with better luminous solar transmittance. Sol. Energy Mater. Sol. Cells.

[33] Okimura K., Mian M.S., Yamaguchi I., Tsuchiya T. (2023). High luminous transmittance and solar modulation of VO_2_-based smart windows with SiO_2_ anti-reflection coatings. Sol. Energ. Mat. Sol. C..

[34] Barimah E.K., Boontan A., Steenson D.P., Jose G. (2022). Infrared optical properties modulation of VO_2_ thin film fabricated by ultrafast pulsed laser deposition for thermochromic smart window applications. Sci. Rep..

[35] Horynová E., Romanyuk O., Horák L., Remeš Z., Conrad B., Peter Amalathas A., Landová L., Houdková J., Jiříček P., Finsterle T. (2020). Optical characterization of low temperature amorphous MoO_x_, WO_x_, and VO_x_ prepared by pulsed laser deposition. Thin Solid Film..

[36] Wang S., Wei W., Huang T., Zhang T., Chen Z., Chen X., Dai N. (2019). Nonstoichiometric oxygen-dependent microstructures and phase transitions in post-annealed vanadium dioxides. Adv. Eng. Mater..

[37] Mahdjoub A., Zighed L. (2005). New designs for graded refractive index antireflection coatings. Thin Solid Film..

